# Potential Role of Birds in the Epidemiology of *Coxiella burnetii*, *Coxiella*-like Agents and *Hepatozoon* spp.

**DOI:** 10.3390/pathogens11030298

**Published:** 2022-02-26

**Authors:** Valentina Virginia Ebani, Francesca Mancianti

**Affiliations:** 1 Department of Veterinary Sciences, University of Pisa, Viale Delle Piagge, 2, 56124 Pisa, Italy; francesca.mancianti@unipi.it; 2 Centre for Climate Change Impact, University of Pisa, Via del Borghetto, 80, 56124 Pisa, Italy; 3 Interdepartmental Research Center “Nutraceuticals and Food for Health”, University of Pisa, Via del Borghetto, 80, 56124 Pisa, Italy

**Keywords:** birds, *Coxiella* *burnetii*, *Coxiella*-like agent, Q fever, *Hepatozoon* spp.

## Abstract

Birds may be involved in the epidemiology of infectious and/or parasitic diseases which affect mammals, including humans. Q fever, caused by *Coxiella burnetii*, is an important zoonosis causing economic losses mainly due to pathologies induced in ruminants. Even though birds are known to be potential reservoirs of *C. burnetii*, their role in the epidemiological cycle of the pathogen is not completely verified. In recent years, new bacteria identified as *Coxiella*-like agents, have been detected in birds affected by different pathologies; the potential role of these bacteria as pathogens for mammals is not currently known. *Hepatozoon* spp. are haemoprotozoa, causing arthropod borne affections within several vertebrate classes. The infection of vertebrate host develops after ingestion of the arthropod final hosts containing oocysts; different tissues and blood cells are then colonized by other parasite stages, such as merozoites and gamonts. In avian hosts, there are several recognized *Hepatozoon* species; however, their life cycle and pathogenicity have not been fully elucidated. Referring to a carrier role by avian species and their ticks in the epidemiology of canine hepatozoonosis, the only clinically relevant affection caused by this parasite genus, they would act as carriers of infected ticks and, when *Hepatozoon americanum* is involved, as paratenic hosts, as well.

## 1. Introduction

Domestic and wild birds can be affected by microorganisms that represent a threat exclusively for their health status, as well as by pathogens transmissible to mammals, including humans. Birds are also regarded as able to transport ixodid ticks, frequently infected with tick borne pathogens and are suspected of dispersal of them in new areas. Climate changes have influenced the epidemiology of several pathogens and contributed to spreading of ticks. Consequently, ticks expanded their geographical distribution and introduced viral, bacterial and parasite agents into new areas [1,2]. Migratory birds fly for long distances and usually arrive in stopover areas where several other animals are present. Pathogens from these zones can successively travel to settlements, such as farms, where domestic mammals occur. Passeriformes and pigeons, but also gulls and waterfowl, are often present in farm areas and can act as a source of infections for livestock, causing relevant economic losses. The role of birds as vectors of bacteria and parasites to farm animals has been attributed to environmental contamination of pasturelands, water supplies and feed by avian feces [3,4,5]. Migratory birds, that fly through traditional and new migratory routes, can carry ticks, posing hazards for human and animal health [6,7].

The aim of the present review was therefore to collect data from the literature about the role of birds in the epidemiology of Q fever that represents a severe threat for the health of humans and other mammals, mainly ruminants. Moreover, the review focused on reports of infections due to *Coxiella*-like agents, new bacterial strains able to cause avian disease, and to *Hepatozoon* spp. in birds, reporting described parasite species and discussing the potential role of birds in spreading of these pathogens.

## 2. *Coxiella burnetii*

### 2.1. Etiology

Coxiella burnetii is a short (0.3–1.0 µm), pleomorphic rod, possessing a membrane similar to Gram-negative bacteria. Even though it was first called *Rickettsia burnetii*, nowadays it is classified as belonging to family *Coxiellaceae* in the order *Legionellales* [8].

It is an obligate intracellular bacterium with two morphologic forms: the small cell variant (SCV) and the large cell variant (LCV), well distinguishable by electron microscopy. *Coxiella burnetii* lives and multiplies in the host’s monocytes and macrophages; as the bacterium multiplies, it destroys the host cell and moves on to live in other cells. LCV replicates inside the acidified auto-phagolysosome and is resistant to lysosomal enzymes and low pH. SCV can persist in the soil free of a eukaryotic host [9]. This form is resistant to heat, cold and many chemical disinfectants. Its resistance is related to a sporulation-like process that protects the bacterium against the external environment, where it can survive for weeks and months [10].

### 2.2. Transmission Routes

*Coxiella burnetii* is the etiologic agent of Q fever, a worldwide zoonosis transmitted by ticks of several species, which release a large number of coxiellae with feces and saliva. However, the infection is usually acquired through inhalation of contaminated aerosol or ingestion of contaminated food, mainly raw milk and dairy products. This pathogen infects a wide range of animal species, including wild and domestic mammals, birds and reptiles. However, the most common reservoirs are cattle, sheep and goats, in which *C. burnetii* causes reproductive disorders. Infected animals may excrete coxiellae mainly in aborted fetuses and vaginal discharge, but also in feces and urine [11].

### 2.3. Coxiella burnetii Infection in Birds

The involvement of avian populations in the epidemiology of *C. burnetii* has been documented since the fifties. Babudieri and Mosovici in 1952 [12] supposed that different bird species could be infected by this pathogen and able to excrete it with their droppings. The authors found seropositive birds, but the most relevant finding was that birds experimentally infected by *C. burnetii* had the pathogen in their excreta. In fact, the agent was found in the kidneys collected from pigeons (*Columba livia*) and sparrows (*Passer italiae*) infected with a *C. burnetii* suspension by oral route. The same authors also reported natural infections in pigeons living in an area where Q fever had affected animals and men [12].

Natural infections were demonstrated by detection of *C. burnetii* in brain and liver of cocks and in shell and shell-membrane of eggs in Turkmenia [13]. *C. burnetii* was also detected in rooks (*Corvus frugileus*) in Russia [14].

Raska and Syrucek [15] examined 572 blood specimens taken from domestic birds in Czechoslovakia in an area of endemic Q fever and found positive reactions to the complement fixation test in hens, turkeys, ducks, geese and pigeons, with hens showing the highest percentage of positive reactions. The same authors confirmed the susceptibility of hens to *C. burnetii* with an experimental infection. Complement fixation tests were also carried out on 480 blood specimens collected from wild birds with positive serological reactions in 15.8% of birds living directly on infected farms, 4.3% of birds living in the immediate vicinity of those farms, and 1.8% of birds living independently of human habitations but in an endemic area. Moreover, during the same study, *C. burnetii* was isolated from the spleen and liver of one redstart (*Phoenicurus phoenicurus*) and one white wagtail (*Motacilla alba*) and from ectoparasites removed from some swallows [15].

In the last two decades, interest in the involvement of birds in the epidemiology of Q fever have significantly increased. Studies based on serological techniques, but mainly on molecular methods, have been carried out on avian specimens and hematophagous arthropods removed from birds.

During a study carried out in Bulgaria with the aim to reveal the contemporary state of Q fever based on vast etiological, clinical and epidemiological investigations testing domestic and wild animals and people, birds were found to be involved in the epidemiology of the disease [16]. *C. burnetii* was considered responsible for several foci in farm animals and in humans in Bulgaria. The investigation revealed that the pathogen was also spread among wild mammals and birds. Serological prevalences of 27.27% in ravens, 26.22% in magpies, 33.33% in pheasants and 11.84% in wood-pigeons were detected. Domestic farm birds were also found to be exposed to *C. burnetii*: the seropositive rate in ducks was 5.75%, in geese 4.09% and in hens 4.19%. The spreading of C. burnetii in wild avian populations was also demonstrated by the isolation of the pathogen from pigeons, turtle doves, crows, ravens and pheasants [16].

In Northern Spain (Basque Country), a PCR investigation on spleen specimens collected from 167 wild birds of different species found *C. burnetii* DNA in a black kite *Milvus migrans* (1 of 7) and in a vulture *Gyps fulvus* (1 of 9) [17]. The observed prevalence was low, but the detection of *C. burnetii* in these avian species was in agreement with other investigations [18,19], in which the authors supposed a relevant role of birds, such as vultures and black kites, in the epidemiology of Q fever because they are often pecking and scraping animal waste or decaying flesh of animal carcasses.

On the other hand, the low prevalence detected in Spain was not in agreement with the results obtained by Ioannou et al. [20], who found a 43% prevalence of specimens scored PCR positive for *C. burnetii* among 131 pools of blood samples collected from 557 wild birds belonging to 51 different species in Cyprus [20]. A very low (0.9%) prevalence was, instead, detected in Slovakia from 2012 to 2013 when blood samples from 347 wild birds were tested by PCR, whereas *C. burnetii* DNA was detected in 2.7% of *Ixodes ricinus* ticks removed from the same analyzed birds [21].

A recent investigation carried out in Russia and Bulgaria found *C. burnetii* DNA in blood and feces of birds, as well as in ticks removed from the same animals [22]. Despite the different prevalence rates, the authors found the Q fever agent in the examined areas of both Russia and Bulgaria. In Russia, bacteremia was detected in 1.4% of the tested birds belonging to the species *Coturnix coturnix* (1/10), *Larus ridibundus* (1/25), *Motacilla alba* (2/52), *Passer domesticus* (1/6) and *Sturnus vulgaris* (1/16). Antibodies against *C. burnetii* antigen were detected in *Anas platyrhynchos* (1/7), Corvus cornix (1/7), Turdus merula (1/6) and Turdus pilaris (3/21). In Bulgaria, 0.5% of the tested birds were found to be bacteremic: *Luscinia luscinia* (3/3) and *Phylloscopus trochilus* (1/4). A higher percentage was found when testing feces. About 14% of the fecal samples were PCR-positive: *Pelecanus onocrotalus* (2/48), *Acrocephalus scirpaceus* (3/16), *Motacilla flava* (1/7), *Turdus merula* (1/3) and *Tringa graleola* (1/7) [22].

Most of the animals whose feces were positive were birds usually living in humid environments. These results are in agreement with those of other studies in which water fowl, in particular *Anas crecca* and *Anas penelope*, were found to be infected by *C. burnetii* [5,23]. The detection of the pathogen in feces of different avian species [24] is relevant, because it shows that infected birds can excrete and disseminate the bacteria in the environment.

Infected waterfowl, in addition to being potential spreaders of coxiellae with their droppings, could be a source of infection for hunters during manipulation and evisceration of the carcasses [23]. Moreover, humid areas are stopover stations where numerous birds, often migratory ones, arrive. Thus, these zones favor the spreading of pathogens, such as *C. burnetii*, that can be transmitted among birds through oral and/or inhalation routes.

Feral pigeons (*Columba livia*) can also be a source of *C. burnetii*. Babudieri and Moscovici [12] proved that these animals may be infected by the Q fever agent, and successively some investigations related foci of human and animal coxiellosis to pigeons.

A study carried out in feral pigeons living in urban areas of Central Italy found *C. burnetii* DNA in 5.95% of spleen specimens [25]. Although the pathogen was detected in spleen samples, pigeons might act as a source of coxiellae for people, as well as their pets, in urban centers.

The possibility of human infections from pigeons has been well documented by Stein and Raoult [26], who described a case of acute Q fever in all five members of one family living on a farm in Provence, in the south of France; the subsequent epidemiological investigation related the outbreak to the exposure to contaminated pigeon feces and ticks [26].

The circulation of *C. burnetii* among feral pigeons was also demonstrated by the detection of antibodies in crop-milk, the caseous material formed by the desquamation of epithelial cells, which parents use for nutrition of the young, from the pigeon crops [27].

Although the role of avian populations in the epidemiology of Q fever has been supposed for several years, few surveys have been carried out to investigate the spreading of *C. burnetii* among birds; thus, information about prevalence rates in different geographic territories are scanty.

The results obtained in the different surveys are difficult to compare because they are strictly in relation to several factors. First of all, they are related to the studied avian population; differences exist between wild and domestic birds, and in this case between animals from farms and those living in captivity as pets. The environment where a given avian species lives influences the spreading of *C. burnetii*; if the pathogen enters into a poultry farm, its spreading is easier than in avian wildlife. Moreover, wild birds living in humid environments, such as waterfowl, could be more frequently infected by this pathogen. Birds, such as pigeons, living in areas with ruminant breeding have more occasion of contracting *C. burnetii*.

The prevalence rates found in the different investigations are also influenced by the test used to detect infected animals. The sensitivity of serological tests employed to detect antibodies in birds is not known; however, it can be influenced by the infection stage. Furthermore, the detection of *C. burnetii* DNA by PCR is related to the employed protocol and the analyzed specimens (feces, blood, spleen); low prevalences of *C. burnetii* detection in birds’ blood have been related to high body temperature that could be a negative factor for the persistence of the bacteremic status [28].

### 2.4. Coxiella burnetii in Hematophagous Arthropods Carried by Birds

*Coxiella burnetii* has been identified in several hematophagous arthropods: lice, mites, parasitic flies and, mainly, soft and hard ticks [29].

Migratory birds carrying *C. burnetii*-infected ticks contribute to the spreading of the pathogen. This issue has been well evidenced by Toma et al. [30], who detected *C. burnetii* DNA in 33% of the analyzed ticks collected from migratory birds in Italy. The pathogen was found in *Haemaphysalis marginatum marginatum*, *H. m. rufipes*, *Hyalomma* spp. and *Ixodes* spp. removed from seven avian species. Two bird species were partial migrants: one common blackbird (*Turdus merula*) and one blackcap (*Sylvia atricapilla*). The remaining five species were long-distance migrants: one common redstart (*Phoenicurus phoenicurus*), two whinchats (*Saxicola rubetra*), two whitethroats (*Sylvia communis*), two nightingales (*Luscinia megarhynchos*) and one European honey buzzard (*Pernis apivorus*) [30].

*Coxiella burnetii* DNA was also detected in *Ixodes ricinus* in Russia, in particular, three nymphs removed from one European robin (*Erithacus rubecola*) [22].

*Ixodes vellantoi* has also been found positive for *C. burnetii*. In fact, the pathogen DNA was detected in a tick of this species removed from a chukar partridge, *Alectoris chukar*, in Cyprus [20].

Ticks parasitizing seabirds seem to be involved in the epidemiology of Q fever as well. An investigation has been carried out on ticks *Amblyomma loculosum* and *Carios capensis* collected from different seabird species in 2011 and 2012 on islands of the Western Indian Ocean [31]. Among the tested *C. capensis* ticks that are typically nest-associated ticks with a strong specificity for seabirds, 65.7% were positive for *Coxiella*. A higher percentage (71.2%) was found in *A. loculosum* ticks that are generalist ectoparasites able to feed on birds, but also reptiles and mammals. Phylogenetic analyses demonstrated that all detected *Coxiella* strains were closely related to *C. burnetii* and demonstrated nearly perfect species specificity for their tick hosts [31].

Although ticks seem to be the main involved arthropods in the spreading of *C. burnetii* among birds, the microorganism has been observed in mites, too.

*Dermanyssus gallinae*, the red poultry mite, is an ectoparasite of poultry, although it frequently bites other birds, such as pigeons, sparrows and pet avian species. *C. burnetii* has been detected in *D. gallinae* removed from nests of canaries, feral pigeons and sparrows [32]. This finding raises an important concern for public health because red mites can also bite humans, which usually causes dermatitis. Avian pets that live in domestic environments, as well as pigeons and sparrows that live in urban areas may be a source of *C. burnetii*-infected mites.

It was previously demonstrated that *C. burnetii* survive about 6 months in live *D. gallinae* and about 1 year in dead red mites [33]. Moreover, as *D. gallinae* is a nidicolous mite, it may also come in contact with coxiellae through contaminated nesting materials such as bird feces. In fact, birds usually remove mites by picking, and in this way, they can ingest *C. burnetii*.

## 3. *Coxiella*-like Infections in Birds

In the last two thousand years, new species of *Coxiella* and closely related proteobacteria have been found in birds and, in some cases, they have been associated with diseases of hosts.

Swainson’s Blue Mountain Rainbow Lorikeets (*Trichoglossus haematodus moluccanus*) were found to be susceptible to these agents. Three lorikeets from a zoo in the U.S. showed lethargy, emaciation and progressive neurological signs and one of them died 24 h after the onset of the symptomatology. During necropsy, hepatomegaly and splenomegaly were observed; moreover, disseminated microgranulomas in liver, spleen and brain other than lymphohistiocytic perivascular encephalitis and cephalic vasculitis were detected by histopathology. Electron microscopy on macrophages in brain lesions detected spherical to rod-shaped prokaryotic organisms having trilaminar cell wall as Gram negative bacteria. Molecular analyses identified the agent as a member of *Coxiella* genus other than *C. burnetii* [34].

Similarly, Shivaprasad et al. [35] diagnosed infection by *Coxiella*-like bacteria in seven psittacine birds and a toco toucan (*Ramphastos toco*). The animals had severe lethargy prior to death. Emaciation and enlarged and mottled pale spleens and livers were macroscopically observed, whereas histology detected multifocal necrosis of hepatocytes with infiltration of macrophages, lymphocytes, plasma cells and heterophils; the spleens had increased numbers of mononuclear phagocytic system cells. Most macrophages had vacuoles with basophilic small cocco-bacilli. The birds were affected by inflammation of epicardium and endocardium, interstitium of the lungs, kidney, adrenal and thyroid glands, lamina propria of the intestine, brain, bursa of Fabricius, and bone marrow; in all the tissues, macrophages containing similar microorganisms were detected [35]. The bacteria were submitted to molecular analyses and on the basis of 16S rRNA gene sequencing, they were identified as members of *Coxiella* genus other than *C. burnetii*, proposed as *Candidatus* Coxiella avium [35].

*Coxiella*-like agents were associated with a systemic infection in a female eclectus parrot (*Eclectus roratus*) that caused the animal death. The infection was characterized by myocarditis, lymphoplasmacytic neuritis, myositis, splenitis, airsacculitis and enteritis [36].

Bacteria belonging to the *Coxiella* genus have also been associated with cases of a disease in blue and yellow macaw (*Ara ararauna*) by Flanders et al. [37]. The animal was lethargic, and cytologic examination of a spleen needle-aspirate identified mononuclear phagocytes with cytoplasmic vacuoles containing structures consistent with bacteria. PCR and sequencing on the splenic specimen detected a *Coxiella*-like agent identical to one previously isolated from the liver of a golden-mantled rosella (*Platycercus eximius*) [35].

More recently, Needle et al. [38] described systemic infections in a black-browed barbet (*Psilopogon oorti*) and a paradise tanager (*Tangara chilensis*). Post-mortem lesions in these animals mainly consisted of meningoencephalitis, hepatomegaly, splenomegaly and interstitial pneumonia; in all the interested organs, bacteria similar to coxiellae were detected by electron microscopy. Also in these cases, molecular analyses classified the agents as *Coxiella*-like organisms.

*Coxiella*-like bacteria were also detected in ticks (*Haemaphysalis wellingtonii*, *H. obesa*, *H. bispinosa*, *Amblyomma testudinarium*) removed from domestic fowls (*Gallus gallus domesticus*), jungle fowls (*Gallus gallus*) and Siamese firebacks (*Lophura diardi*) in Thailand [39].

*Coxiella*-like organisms were found in *C. capensis* and *Ixodes uriae* collected from seabirds during 2003–2013 from distant geographic locations covering their entire distribution, including circumpolar areas of both hemispheres, as well as temperate and tropical areas of the Pacific, Atlantic and Indian Oceans [40].

*Argas monolakensis*, a bird feeding tick native to Mono Lake California (USA), was found to harbor a *Coxiella* sp., which is similar to *C. burnetii* and *Coxiella cheraxi*, a crayfish pathogen, but it is not clear if this strain is an endosymbiont of ticks or an animal pathogen. The potential pathogenicity of this strain could be verified, because *A. monolakensis* usually feeds on gulls (*Larus californicus*), but sometimes on humans, too [41].

An undescribed *Coxiella* sp. was also detected in *C. capensis* ticks collected from nests of brown pelicans (*Pelecanus occidentalis*) on Deveaux Bank Heritage Preserve, U.S. Amplification of this *Coxiella* sp. DNA from all ticks, including egg masses and unfed larvae, provides evidence of transovarial and transstadial transmission of the agent [42]. This novel agent could be an endosymbiotic *Coxiella*; however, the nature of the relationship between bacterium and tick host, as well as the potential pathogenicity of this *Coxiella* sp. for vertebrates, have not been clarified. Table 1 reports the avian species in which *Coxiella*-like agents have been detected.

## 4. *Hepatozoon* Species

### 4.1. Etiology

*Hepatozoon* spp. (Phylum Apicomplexa belonging to subgenus Adelorina, Family Hepatozooidae) infect blood and tissues of many vertebrates, including reptiles and amphibians [43]. Their life cycle consists of merogony and gametogony in a vertebrate host, while syngamy and sporogony occur in haematophagous invertebrates, usually ticks, acting as definitive hosts. Ixodidae and Argasidae are involved, as well as insects such as Anoplura, mosquitoes, *Glossina* spp., fleas and triatominae [44].

In vertebrate hosts merogony occurs in different tissues (liver, spleen, bone marrow, lymph nodes, intestine), then gamonts can parasitise erythrocytes and/or, more frequently, leucocytes in birds and mammals [45]. However, a complete and documented life cycle is available for only a few of the parasite species [43].

*Hepatozoon* are considered paraphyletic, and the taxonomy should be revised [46,47]. Within the genus occur a monophyletic group (*Hepatozoon* sensu stricto), which includes *H. canis, H. felis* and *H. americanum* [47] infecting and causing disease in carnivores. Conversely, the taxonomy of *Hepatozoon* sensu lato is unsettled, and different phylogenetic clades are defined as “evolutionary units deserving the status of separate genera” [44].

### 4.2. Transmission Routes

The vertebrate usually becomes infected by ingestion of the cyclic vector. However, paratenic hosts seem to occur in some *Hepatozoon* sp. life cycle from reptiles [47,48,49] and have also been reported in the canine parasite *H. americanum* [50]. Vertical transmission has been described in reptiles [51].

### 4.3. Hepatozoon spp. in Birds

Avian *Hepatozoon* spp. usually parasitize mononuclear leucocytes. *Hepatozoon peircei* and *Hepatozoon ellisgreineri* were reported to colonize red blood cells and granulocytes (mostly heterophils), respectively, widely differing from all other blood apicomplexas in birds [52,53]. However, birds affected by *Hepatozoon* rarely show clinical signs, parasitaemias are low and good morphologic criteria and data about the life cycles are lacking. Blood stages of *Hepatozoon* are morphologically very similar to other protozoa belonging to the same order, such as *Lankestrella* [53]. This organism in fact can be found in the same types of host cells [54].

The taxonomy of *Hepatozoon* in avian species has not been fully clarified. Studies until now have mostly been based on microscopic features of parasites. At present, 20 species have been identified (Table 2), but among them, *Hepatozoon parus* and *Hepatozoon kaabeni*, after molecular studies, appear more similar to the amphibian parasite *Lankestrella* [55,56], although a possible co-infection with another apicomplexan in examined birds could not be ruled out [53]. These findings have been recently confirmed, after a morphobiologic and molecular study showing the lack of syzygy and sexual stages in invertebrate hosts, so allowing the repkacement of former *H. kaabeni* with *Lankestrella kaabeni* [54].

Future trends mostly consist of molecular and morphologic characterization of avian hepatozoa, along with the attempts of transmission studies.

Hepatozoonoses in birds are regarded as not serious illness, except for an outbreak of fatal hepatozoonosis in young captive *Grus monacha* from Japan [65].

Avian hepatozoa have not been studied in depth, and the parasite vectors are unknown [53,56], although Bennet et al. [57] suggested an argasidae (*Ornithodoros peringueyi*) and a flea (*Xenopsylla trispinis*) as possible vectors for *Hepatozoon atticorae.* However, *Hepatozoon* species infecting mammals and birds would show different specificity for their final hosts [43].

### 4.4. Hepatozoon spp. in Ticks Infesting Birds

Birds may carry hard ticks, mostly in their early stages (larvae and nymphs), contributing to dispersal of these acarine, along with several tickborne pathogens. This feature has been reported mostly in passerine and in ground and shrub dwelling birds as well as potentially migratory ground feeding avian species [66].

*Hepatozoon* spp. are not zoonotic organisms; nevertheless, canine hepatozoonosis may represent a threat for dogs. *Hepatozoon canis* and *H. americanum* have been described with geographical and morphobiological differences. The infection of canine host by both species starts by ingestion of a tick containing oocysts. Sporozoites of *H. canis* colonize the lymph nodes, spleen and bone marrow, while *H. americanum* can be found in monocytes between fibers of scheletric and cardiac muscles. For this latter parasite, species infection also occurs for ingestion of paratenic hosts. Disease caused by *H. canis* can develop as subclinical to life threatening depending on the host immune status or coinfections occurring. Although to date there is no evidence to link birds to the epidemiology of canine *Hepatozoon* spp., this could be a future research interest, considering the biology of Ixodidal final hosts involved, as well the potential of birds to act as paratenic hosts for *H. americanum*. As a matter of fact, the main arthropod vectors for these parasite species are *Rhipicephalus sanguineus* and *Amblyomma ovale* (*H. canis*), while *Amblyomma maculatum* would transmit *H. americanum* [67,68,69,70]. However, *H. canis* DNA was found in *Dermacentor* spp. [71,72], in *Haemaphysalis* species, namely *Haemaphysalis concinna* [71,73], *Haemaphysalis longicornis* [74] and *Haemaphysalis punctata* [73], while the vectorial competence has been ruled out for *I. ricinus* [75]. Furthermore, *H. longicornis* has been recently reported as a spreading tick [76].

All these ixodid species can feed on avian hosts. Furthermore, although birds are unusual hosts for *R. sanguineus* [77], the bird delivery of this tick species has not been excluded [78], while wild birds play a not secondary role in the biology of *A. ovale* [79].

Birds are considered as hosts of *A. maculatum* immature stages, and their role in the maintenance of *H. americanum* and in its life cycle has not been fully clarified [80]. Moreover, larval stages of the tick can be infected with *H. americanum*, and nymphs are infective for dogs, as well as adults, unlike *H. canis* [80]. Dogs can acquire the infection by ingesting adult infected ticks and by predation of rodents or birds harboring the larval or nymphal tick stages [81].

Transovarial transmission of *H*. *canis* does not occur [68,82], and this route is neither suspected in *H. americanum* life cycle, nor for other definitive hosts of *Hepatozoon* spp. [83]. However, the transstadial transmission, would allow for a wider range of intermediate hosts, mostly for *H. americanum.*

## 5. Conclusions

Q fever is an important zoonosis causing problems for the mammals’ health and economic losses in farms. In view of the threats related to this disease, it is important to know all the possible sources of infection and the routes of transmission.

Even though *C. burnetii* has been found in different avian species in several studies, birds are not always considered as a possible source of this agent when Q fever foci occur. Instead, hygienic prophylaxis, mainly in farms, must also pay attention to domestic and wild birds which can come in contact with mammals, directly or through their droppings.

In the last years, the detection of *Coxiella*-like microorganisms in birds showing diseases, as documented by clinical signs and pathological findings, shows that new pathogens, not yet well known, can affect avian populations. Further studies are necessary to better understand the pathogenicity of these bacteria for avian species, mainly for poultry and aviary birds. Studies should also be performed to evaluate the pathogenicity of *Coxiella*-like bacteria for humans and other mammals and the routes of transmission.

Avian hepatozoonosis is a not well-known parasitic disease; *Hepatozoon* spp. affecting birds have not completely been described, definitive host species have not fully recognized, and the taxonomic status of some parasites is still debated. Furthermore, in addition to the low host specificity of most etiologic agents of hepatozoonoses, these infections cannot be regarded as zoonoses. For these reasons, avian species could take on a role in canine hepatozoonosis, acting as carrier of infected ticks and, when *H. americanum* is involved, as paratenic hosts, too. In conclusion, although *Hepatozoon* is widely distributed among all groups of tetrapods and birds, systematic and molecular studies are needed to achieve full knowledge of avian hepatozoonosis.

## Figures and Tables

**Table 1:** Bird species in which *Coxiella*-like agents have been detected in post-mortem specimens.

Bird Species	References
Swainson’s Blue Mountain Rainbow Lorikeets (*Trichoglossus haematodus moluccanus*)	[34]
Toco toucan (*Ramphastos toco*)	[35]
Psittacine birds	[35,38]
Eclectus parrot (*Eclectus roratus*)	[36]
Blue and yellow macaw (*Ara ararauna*)	[37]
Black-browed barbet (*Psilopogon oorti*)	[38]
Paradise tanager (*Tangara chilensis*)	[38]

**Table 2:** Avian *Hepatozoon* species.

Bird Family	Parasite Species	References
Accipitridae	*Hepatozoon neophrontis*	[57]
Sagitariidae	Hepatozoon ellisgreineri *	[53]
Apterygidae	*Hepatozoon kiwii*	[58]
Diomedeidae	*Hepatozoon albatrossi*	[59]
Emberizidae	*Hepatozoon paroariae*	[57]
Thraupidae	*Hepatozoon rhamphocoeli*	[57]
Estrildidae	*Hepatozoon estrildus*	[57]
Hirundinidae	*Hepatozoon atticorae*	[60]
Laniidae	*Hepatozoon lanis*	[57]
Ianiidae	*Hepatozoon malacotinus*	[57]
Sylviidae	*Hepatozoon sylvae*	[57]
Paridae	*Hepatozoon parus§*	[55,60]
Passeridae	*Hepatozoon passeris*	[61]
Numididae	*Hepatozoon numidis*	[57]
Pittidae	*Hepatozoon pittae*	[57]
Prionopidae	*Hepatozoon prionopis*	[62]
Acrocephalidae	*Hepatozoon kabeeni ^§^*	[54,56,63]
Apodidae	*Hepatozoon apodis*	[64]
Zosteropidae	*Hepatozoon zosteropis*	[57]
Hydrobatidae	Hepatozoon peircei **	[52]

Legends—* Within granulocytes; ** within erythrocytes; § *Lankestrella* spp.

## Data Availability

The data presented in this study is contained within the article.
